# Exploring Pharmacological Mechanisms of Xiang Ju Tablets in the Treatment of Allergic Rhinitis via a Network Pharmacology Approach

**DOI:** 10.1155/2019/6272073

**Published:** 2019-09-12

**Authors:** Kun xia Hu, Xi Duan, Li zhu Han, Hong ye Ju, Bin Wang, Zhi shu Tang, Xiao Song

**Affiliations:** ^1^College of Pharmacy, Shaanxi University of Chinese Medicine, Xianyang, Shaanxi 712046, China; ^2^Affiliated Hospital, Shaanxi University of Chinese Medicine, Xianyang, Shaanxi 712000, China

## Abstract

In this study, allergic rhinitis (AR) disease targets and Xiang Ju tablet-associated targets were determined through the use of databases for the identification of putative therapeutic targets and then combined. After the production of a putative therapeutic target interaction network for Xiang Ju tablets against AR, topological analysis was used to determine the core targets of Xiang Ju tablets in AR treatment. For all putative therapeutic targets, analyses of biological function and pathway enrichment were performed to optimize the biological processes and key signaling pathways of Xiang Ju tablets in AR treatment. The top 5 therapeutic targets of Xiang Ju tablets in AR treatment were identified and included CXCL8, IL1B, IL6, IL10, and TNF. The biological processes, molecular functions, and cell composition related to the use of Xiang Ju tablets in AR treatment were predominantly associated with cytokine production, regulation of protein secretion, and regulation of peptide secretion; cytokine activity, cytokine receptor binding, and receptor ligand activity; and platelet alpha granule lumen, collagen-containing extracellular matrix, and platelet alpha granule. In addition, the top 64 key signaling pathways were identified.

## 1. Introduction

Allergic rhinitis (AR) is a very common disease that is most prevalent in children and adolescents. AR is spreading rapidly worldwide and affects approximately 40% of the population [1, 2]. AR is characterized by an imbalance of T-helper type 1/T-helper type 2 (TH1/TH2) cells and a deficiency of regulatory T cells [3]. The typical symptoms of AR are paroxysmal sneezing, running nose, nasal obstruction, rhinocnesmus, and partly accompanied by olfactory drop [4]. Complications of AR include asthma, conjunctivitis, chronic rhinosinusitis, adenoid hypertrophy, and secretory otitis media [5]. In addition, the presence of AR can aggravate asthma, and most patients with asthma also have AR [6]. At present, the treatments for AR are mainly drug therapy, including antihistamines, glucocorticoids, antileukotrienes, ketones, nasal decongestants, nasal anticholinergic drugs, and traditional Chinese medicine (TCM). However, their therapeutic effects against AR are not ideal; therefore, new and effective remedial measures must be developed to treat AR, and clarifying the potential mechanism of AR would facilitate finding more suitable methods to treat AR.

TCM has been widely used for the prevention and treatment of various diseases for thousands of years, mainly in China and the surrounding areas. TCM has increasingly attracted worldwide attention from clinicians and researchers, owing to its demonstrated efficacy, broad applications, and less side effects [7]. In accordance with the standards (YBZ12122004) of the state food and drug administration (trial), Xiang Ju tablets are made by decocting and extracting *Platycarya strobilacea* Sieb. et Zucc (with seeds removed), Prunellae Spica, Chrysanthemi Indici Flos, Astragali radix, Magnoliae Flos, Saposhnikoviae Radix, Angelicae dahuricae radix, Glycyrrhizae radix et rhizoma, and Chuanxiong Rhizoma. The Xiang Ju tablets have a Xinsan Qufeng, Qingre Tongqiao effect, on the treatment of acute and chronic sinusitis rhinitis [8]. Studies have found that Xiang Ju tablets are used in the treatment of chronic rhinitis sinusitis and allergic rhinitis, especially acute paroxysmal rhinitis, because of significant efficacy and fewer side effects [8, 9]. In case of ineffective treatment, the patient can be treated by prolonging the time of administration of Xiang Ju tablets or adding antibiotics.

Network pharmacology, proposed by Hopkins in 2007, is used to elucidate drugs' effects on multiple targets [10]. It can be applied to TCM theory to highlight holistic thinking regarding multilevel interactions among herbs, targets, and diseases [11, 12]. Network pharmacology is an emerging field that integrates multiple disciplines and techniques such as genomics, topology, and computational omics and attempts to explore potential mechanisms and relationships by constructing multiple network models. With the rapid development of bioinformatics, network pharmacological methods have become a new means to efficiently and systematically explore the safety of TCM formulations or the molecular mechanisms of multidrug combinations and have provided a new approach for drug mechanism research and drug development [13, 14]. Network pharmacology, one of the emerging disciplines, has incomparable advantages over traditional methods in elucidating the comprehensive mechanism [15]. It has been successfully applied in TCM-related research fields for the elucidation of therapeutic mechanisms. For example, Xie et al. have applied network pharmacological analysis to explore the mechanism of action of Radix Astragali Angelica in traumatic brain injury [16]. Li et al. have discovered the protective mechanism and main bioactive compounds of Gualou Xiebai decoction in coronary heart disease through network pharmacological analysis [17]. Li et al. have explored the antimyelofibrosis mechanism of Radix Salviae through network pharmacology [18].

**Figure 1:**
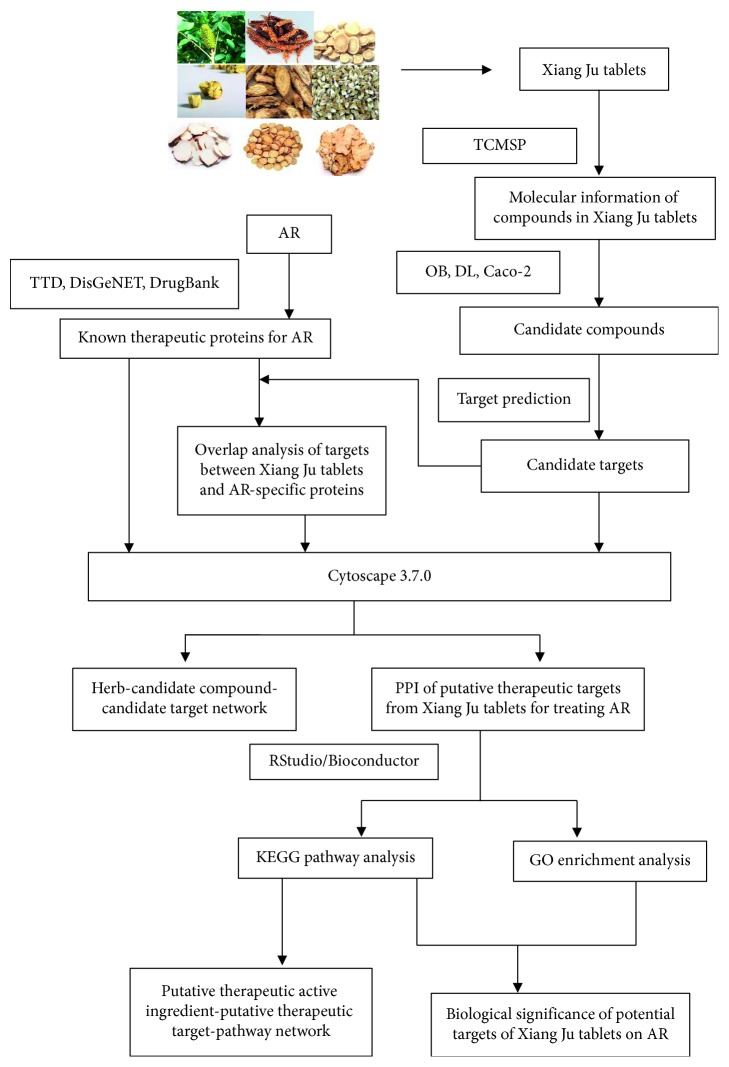
Schematic diagram of the pharmacological mechanism of prescribed Xiang Ju tablets for the treatment of AR, based on network pharmacology.

In this study, network pharmacology was used to establish a herb-compound-target-pathway network to explore the mechanism of Xiang Ju tablets in treating AR. The flowchart of the experimental procedures of our study is shown in Figure 1.

## 2. Materials and Methods

### 2.1. Data Preparation

#### 2.1.1. Chemical Ingredient Database Building

To build a database of the compounds in Xiang Ju tablets, we used the Traditional Chinese Medicine Systems Pharmacology Database [19, 20] (TCMSP; http://lsp.nwu.edu.cn/tcmsp.php), a unique system pharmacology platform designed for Chinese herbal medicines and containing information on their absorption, distribution, metabolism, and excretion (ADME) characteristics, targets, related diseases, and pathways [21].

#### 2.1.2. Pharmacokinetic Prediction

Xiang Ju tablet active compounds were screened mainly on the basis of oral bioavailability (OB), drug likeness (DL), and Caco-2 permeability (Caco-2), the three most important indicators for bioinformatic evaluation of ADME characteristics. Specifically, the ingredients meeting the criteria of OB ≥ 30%, DL ≥ 0.18, and Caco-2 ≥ 0.4 were chosen as candidate compounds for further analysis [22, 23].

OB, the rate and percentage of an oral dose of a drug that is absorbed into the blood circulation and produces pharmacological effects, is one of the most important pharmacokinetic characteristics of oral drugs. It reflects the effectiveness of oral drugs in entering human circulation, which is particularly important in drug discovery of TCM for most oral Chinese herb formulas [24]. In this study, OB ≥ 30% was regarded as a threshold for screening possible candidate drugs.

In the early stages of drug development, the evaluation of DL aids in screening excellent compounds [25]. DL is a qualitative profile used in drug design to evaluate whether a compound is chemically suitable for use as a drug and how drug-like a molecule is with respect to the parameters affecting its pharmacodynamic and pharmacokinetic profiles, which ultimately affect its ADME properties [26].

Caco-2 cell monolayers are widely applied as a standard permeability-screening assay for predicting a compound's intestinal absorption and the fraction of the oral dose absorbed in humans [27]. The Caco-2 cell permeation values of all molecules are calculated with an in silico model by using the VolSurf approach [13].

#### 2.1.3. Potential Targets of Xiang Ju Tablet Chemical Components

According to TCMSP, the effective compounds in Xiang Ju tablets were matched with potential targets one by one, and the target species was selected as “humans” through the UniProt database (http://www.uniprot.org/) containing the predicted targets of candidate compounds in Xiang Ju tablets.

#### 2.1.4. AR Significant Targets

We used Therapeutic Targets Database v4.3.2 [28] (https://db.idrblab.org/ttd/), DrugBank v5.1.2 (https://www.drugbank.ca), and DisGeNET v6.0 [29] (http://www.disgenet.org) database retrieval to obtain targets related to AR and applied the UniProt database (https://www.uniprot.org) to disease targets for standardization. We determined the UniProt accession numbers and gene symbols.

### 2.2. Protein-Protein Interactions

In order to clarify the interaction between AR-related targets and potential targets of Xiang Ju tablets, we intersected the disease targets and drug targets and input the disease target and drug component targets into Venny 2.1 (http://bioinfogp.cnb.csic.es/tools/venny/index.html) software to obtain the target intersection. The selected common target proteins of diseases and drug components were used to construct the protein-protein interaction (PPI) network model on the STRING v11.0 [30] (https://string-db.org/) platform. We set the protein type to “*Homo sapiens*” and left the default settings in place for other parameters. We then exported the “string_interactions.tsv” file and imported it into Cytoscape 3.7.0 [31, 32] to obtain the PPI network.

### 2.3. Gene Ontology and Pathway Analysis

Fisher's exact test method was used. The data package was clusterProfiler from RStudio/Bioconductor [33]. The selected standard *p* value ≤0.05 and *q* value ≤0.01 were arranged in the descending order according to the value of the enrichment factor, and the results were plotted.

### 2.4. Network Construction

Five types of networks: herb-compound-target-AR-related target network, putative therapeutic component-putative therapeutic target network, protein-protein interaction network, core putative therapeutic target network, and putative therapeutic component-putative therapeutic target-pathway network, were generated to decipher the mechanisms of Xiang Ju tablets in the treatment of AR. Cytoscape 3.7.0 [34, 35] software was used to construct the network. Network Analysis, a plugin in Cytoscape 3.7.0, was used to analyze the topological properties of the network.

## 3. Results

### 3.1. Drug Compound Information

We obtained 976 components from Xiang Ju tablets. OB, DL, and Caco-2 thresholds were used to eliminate the compounds with duplicates and the compounds without target prediction data. The 145 active compounds from the 9 traditional Chinese medicines found in Xiang Ju tablets were searched in domestic and foreign literature databases and the Traditional Chinese Medicine Systems Pharmacology Database. In addition to the above 133 molecules, 12 ingredients with low OB, DL, or Caco-2 were considered active components, owing to their reported treatment effects, e.g., ferulic acid, which has been found to exert potent immunomodulatory effects against intestinal inflammatory responses in a mouse model. These compounds were also selected for further study because of their good pharmacological effects. In brief, 145 ingredients from Xiang Ju tablets were selected for further research analysis, including 6 in *Platycarya strobilacea* Sieb. et Zucc, 6 in Prunellae Spica, 11 in Astragali radix, 5 in Chrysanthemi Indici Flos, 12 in Saposhnikoviae Radix, 14 in Magnoliae Flos, 71 in Glycyrrhizae radix et rhizoma, 7 in Chuanxiong Rhizoma, and 13 in Angelicae dahuricae radix, which were collected from TCMSP.

### 3.2. Target Prediction of Xiang Ju Tablets

A total of 330 potential targets from the 145 compounds were generated by using the target prediction model. The amount of potential target hits for the *Platycarya strobilacea* Sieb. et Zucc, Prunellae Spica, Astragali radix, Chrysanthemi Indici Flos, Saposhnikoviae Radix, Magnoliae Flos, Glycyrrhizae radix et rhizoma, Chuanxiong Rhizoma, and Angelicae dahuricae radix drugs were 194, 213, 200, 208, 78, 34, 232, 43, and 61, respectively. We obtained 170 targets related to AR through the Therapeutic Targets Database and DrugBank and DisGeNET databases.

### 3.3. Network Construction and Analysis

**Figure 2:**
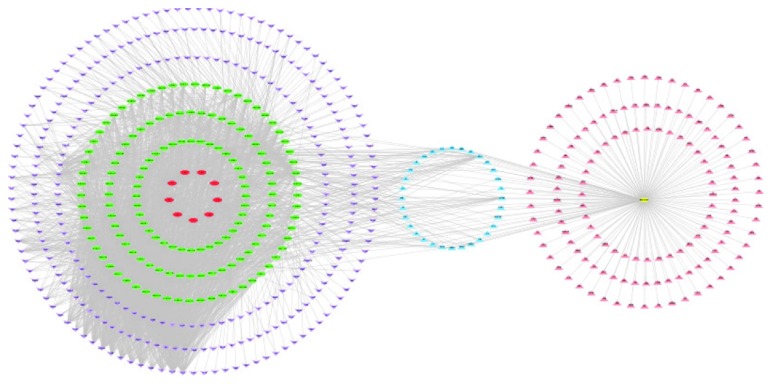
Herb-candidate compound (Cc)-candidate target (Ct)-AR-AR-related target (AR-rt) network for Xiang Ju tablets (red nodes represent the herbs, green nodes represent the candidate compounds, purple nodes represent the candidate targets, blue nodes represent the candidate target intersection, yellow nodes represent the AR, and pink nodes represent the AR-related targets).

We established a network through network analysis to elucidate the relationships among the herbs, candidate compounds, candidate targets, AR, and AR-related targets (Figure 2). This network consisted of 623 nodes (9 herbs, 145 candidate compounds, 298 candidate targets, 32 putative therapeutic targets, 1 AR, and 138 AR-related targets) and 2783 edges. We found that the higher the value of the node degree and median centrality, the more critical the corresponding compound or target in the network. In the Xiang Ju tablet active ingredient-forecast target network, the targets had an average value of 8.93, and the top 20 targets interacted with more than 45 compounds, with a single molecular function in multiple target proteins and multiple molecular phenomena on the same target protein, in accordance with TCM composition and the features of target interaction.

### 3.4. Putative Therapeutic Target Interaction Network

**Figure 3:**
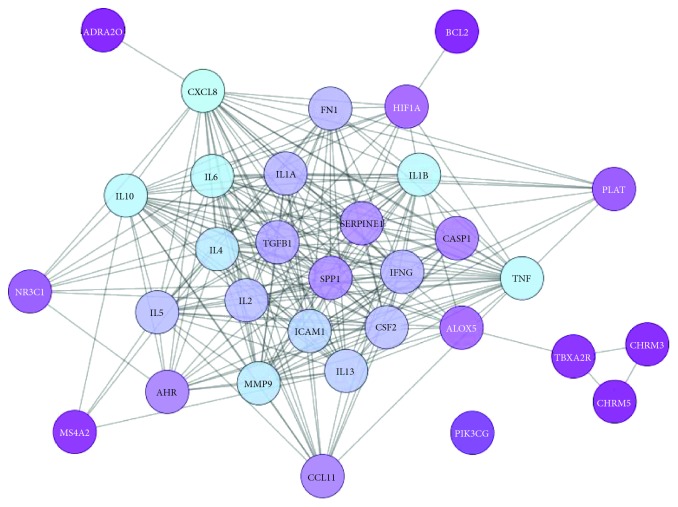
AR-related putative therapeutic target protein interaction network: PPI network of targets for Xiang Ju tablets against AR (the closer the node color is to blue, the higher the degree).

The network relationship and target prediction of TCM in treating diseases can be achieved by using online databases and relevant software. In this study, the network document of “candidate compounds-candidate targets-AR-related targets” was imported into Cytoscape 3.7.0 software to identify 32 putative therapeutic targets of Xiang Ju tablets in treating AR. The intersection of the target of components in Xiang Ju tablets and the AR targets was obtained. The 32 obtained putative therapeutic targets (Table 1) were imported into the STRING database to establish the putative therapeutic target protein interaction network (Figure 3). The network had 32 nodes, which interacted with 236 edges. From purple to blue, the degree of freedom is increasing, and thicker edges indicate the stronger interactions. Our results together indicated that the top mutual target proteins have various beneficial functions to treat AR at the molecular level.

In this network, the degree of blueness of the nodes (CXCL8, IL1B, IL6, IL10, TNF, MMP9, IL4, and ICAM1) is high, and the number of edges (the number of gene nodes that they are associated with) of each node is quite large (25 in CXCL8; 24 in IL1B, IL6, IL10, and TNF; 23 in MMP9 and IL4; 22 in ICAM1). These results demonstrate that these genes are closely related to other genes in the network and consequently may play an important role in AR. IL8 (CXCL8) attracts and activates polymorphonuclear cells in sinonasal mucosa, and it can induce granulocyte recruitment [36]. Several studies have demonstrated that TNF enhances the effect of IL4 on IgE production; TNF also has an important role in the expression of adhesion molecules that induce transendothelial migration of eosinophils [3]. IL10 is a cytokine produced by a number of immune cells, including T cells, B cells, macrophages, and monocytes [37]. Allergen-specific immunotherapy has been shown to induce allergen-specific immune tolerance, most probably through the upregulation of IL10 and downregulation of IL4 [38]. Intercellular adhesion molecules have been found to play crucial roles in the pathogenesis of AR [39]. In a murine model of AR, subepithelial fibrosis and gland hypertrophy have been found to be associated with increased expression of MMP9 [40].

### 3.5. Putative Therapeutic Compound-Putative Therapeutic Target Network Construction

**Figure 4:**
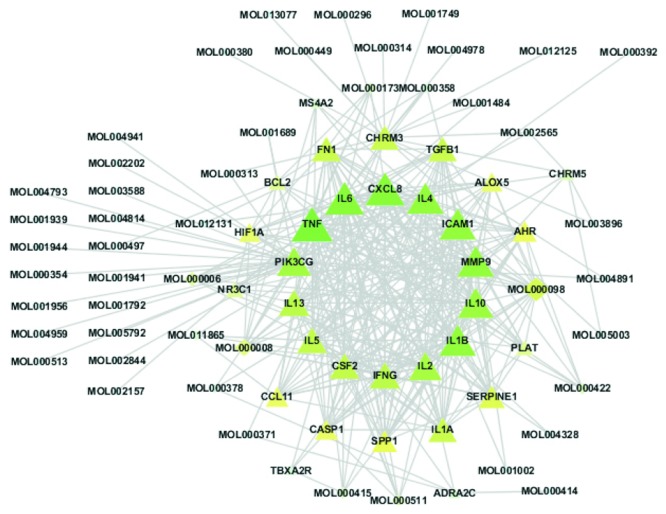
Putative therapeutic component-putative therapeutic target network diagram (diamond represents the presumed therapeutic active ingredient, and triangle represents the presumed therapeutic target).

The Merge function of Cytoscape 3.7.0 software was used to combine the candidate compound-candidate target network and the putative therapeutic target interaction network, and 48 compounds that play a role in treating AR in the composite were obtained (Table 2). The visual interactive network of “putative therapeutic components-putative therapeutic targets” in the composite was constructed (Figure 4). The targets of 48 putative therapeutic components in the treatment of allergic rhinitis include IL6, TNF, CXCL8, ICAM1, ILA, MMP9, IL10, and IL1B.

In previous studies, quercetin has been found to inhibit mast cell activation, inflammatory cytokine production, and histamine release after immunological stimulation. Quercetin may suppress AR in rats by increasing Cl transport and ciliary beat frequency [41]. Quercetin modulates the activation of inflammatory cells and neuropeptide production and results in improvement of clinical conditions of allergic diseases, especially AR [42]. Kaempferol can significantly decrease the early-phase response to allergen exposure (such as from nasal rubs), IgE production, and histamine release, as well as late-phase responses such as expression of inflammatory markers (IL32, TSLP, IL4, IL8, ICAM1, MIP-2, and COX-2). Kaempferol also decreases caspase-1 activation and can aid in treating allergic inflammatory diseases, including AR [43]. The phenolic acids and flavonoids in *Xanthii fructus* contribute to the main anti-inflammatory effects. According to spectral correlation theory, the relationship between the chemical composition of *Xanthii fructus* and its effect on AR has been described by using the co-correlation coefficient, and apicin has been confirmed to be the main active compound of Magnoliae Flos [44].

### 3.6. Screening of Core Therapeutic Targets

**Figure 5:**
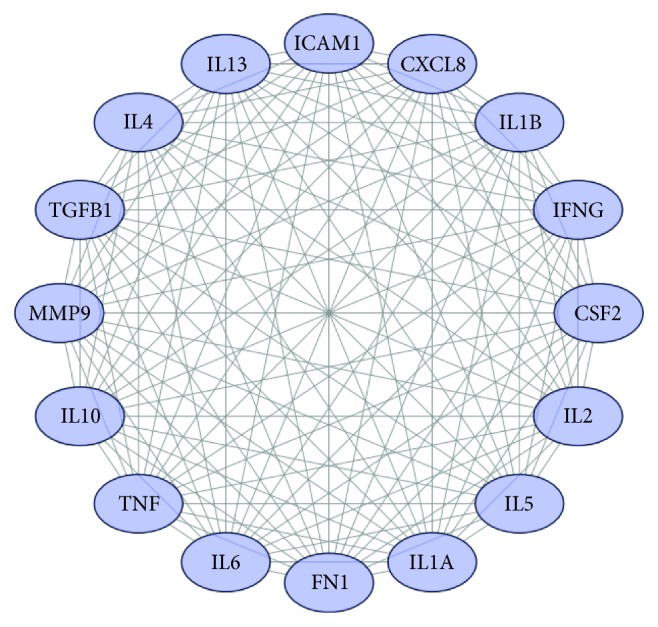
Network of the top 16 core therapeutic targets.

Data analysis of the network topology showed that the maximum degree of freedom and the mean degree of freedom were 25 and 14.75, respectively. The network of the top 16 core therapeutic targets was constructed, and the graphic visualization was performed according to the degree of node freedom and the edge thickness (Figure 5). The degrees of the 16 candidate targets were all 15. Sixteen core therapeutic target proteins were identified: CXCL8, IL1B, IL6, IL10, TNF, MMP9, IL4, ICAM1, IL13, CSF2, IL2, IL5, IFNG, TGFB1, FN1, and IL1A.

### 3.7. Gene Ontology (GO) Functional Enrichment Analysis

**Figure 6:**
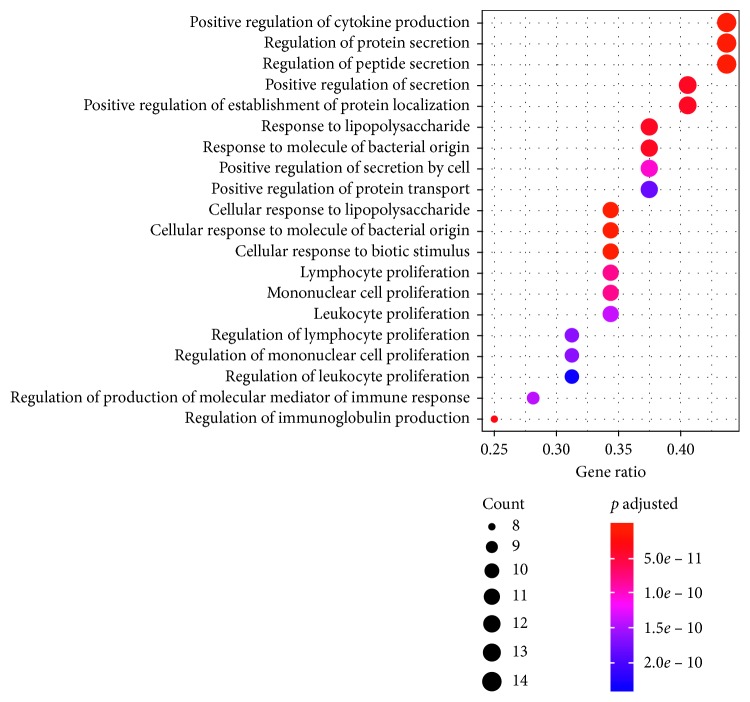
GO BP enrichment analysis of the putative therapeutic targets of Xiang Ju tablets in the treatment of AR (*p* value <0.05; *q* value <0.01). The *y*-axis shows significantly enriched GO BP of the target genes, and the *x*-axis shows the gene ratio (the ratio of the number of target genes belonging to a pathway to the total number of annotated genes located in the pathway). A higher gene ratio represents a higher level of enrichment. Dot size indicates the number of target genes in the pathway, and dot color reflects the *p* adjusted range.

**Figure 7:**
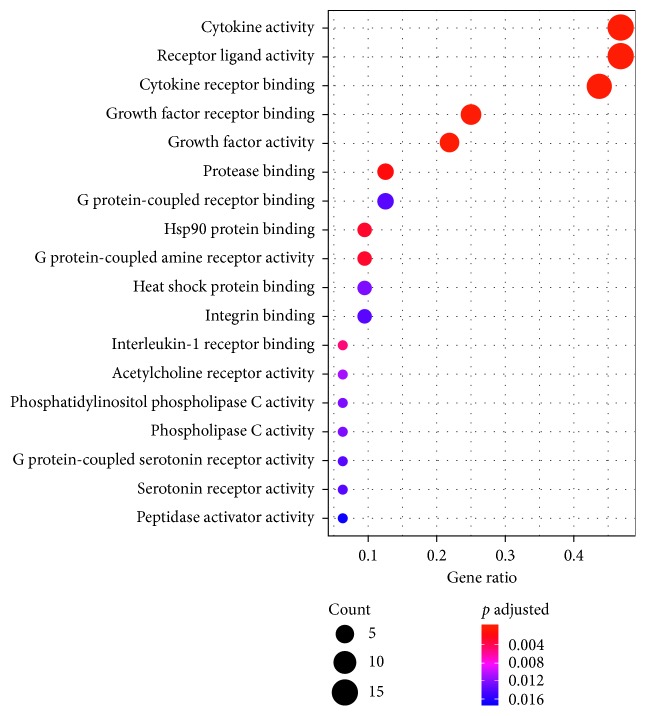
Molecular function analysis of putative therapeutic targets.

To further clarify the biological effects involved in the treatment of AR with Xiang Ju tablets, we performed GO analysis of AR-related putative therapeutic targets. GO annotation and enrichment of genes encoding Xiang Ju tablet protein targets were conducted from three aspects: biological process (BP), molecular function (MF), and cell composition (CC). We identified 937 enrichment results in the related items of biological process, which included positive regulation of cytokine production, regulation of protein secretion, regulation of peptide secretion, and other biological processes. Eighteen enrichment results were related to molecular function, including cytokine activity, cytokine receptor binding, receptor ligand activity, and other molecular functions; three enrichment processes were related to cell composition, including platelet alpha granule lumen, collagen-containing extracellular matrix, and platelet alpha granule. We selected the 18 items of molecular function and the first 20 items of BP and then performed GO enrichment analysis in RStudio software (Figures 6 and 7).

### 3.8. Pathway Analysis

**Figure 8:**
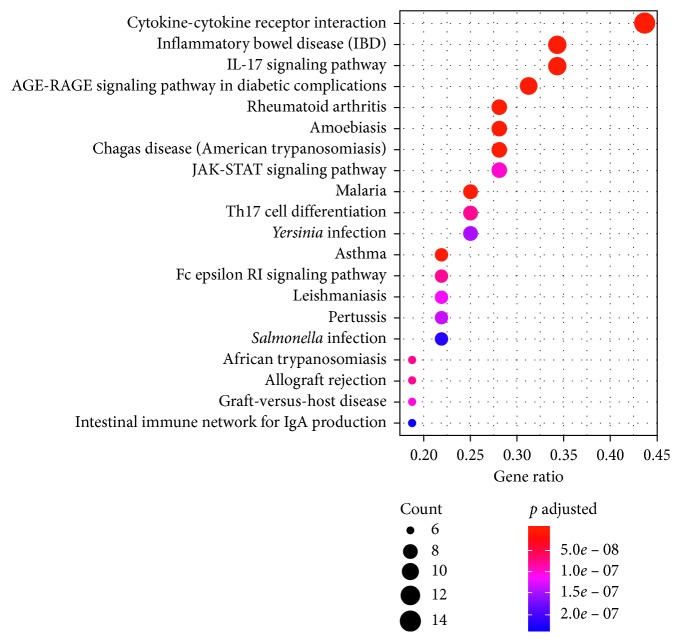
KEGG enrichment pathway analysis of putative therapeutic targets.

To clarify the biological actions of these targets, we performed GO analysis and pathway enrichment analysis on the basis of the RStudio (Bioconductor) database. We input a gene list of major AR-related putative therapeutic targets into RStudio, generating relevant pathways that might have an important influence on the biological process of Xiang Ju tablets in treating AR. Only pathways with a *p* value <0.05 were considered significant. A total of 64 KEGG signaling pathways were obtained through pathway enrichment analysis, 57 of which were significantly enriched. In the bubble diagram (Figure 8), the color and size of the nodes were determined according to the number and *p* value of related genes. The color from blue to red reflects *p* values from large to small, and node size indicates the number of related genes. Inflammatory bowel disease, the IL17 signaling pathway, cytokine-cytokine receptor interaction, the AGE-RAGE signaling pathway in diabetic complications, malaria, and the asthma pathway were found to be induced.

The inflammation response plays an important role in host survival and also leads to acute and chronic inflammatory diseases such as rheumatoid arthritis, bowel diseases, AR, asthma, atopic dermatitis, and various neurodegenerative diseases [45]. IL17, a key cytokine produced by Th17 cells, induces allergen-specific Th2 cell activation, eosinophil and neutrophil accumulation, and serum IgE production in asthma; all these features may play important roles in AR [46]. AGE-RAGE regulates different signaling pathways; for example, AGE combined with RAGE activates downstream signals through PKC, such as p38, MAPK, TGF-*β*, and NF-*κ*B. AGE-RAGE has been found to promote the occurrence and development of various diseases, owing to its influence on many downstream genes as well as signaling pathways [47].

### 3.9. Target-Pathway Network Analysis of Xiang Ju Tablets

**Figure 9:**
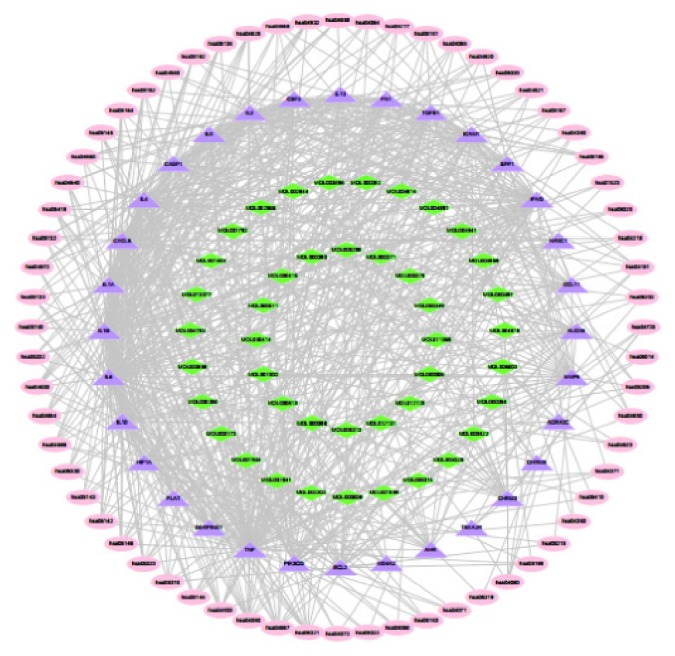
Putative therapeutic active ingredient-putative therapeutic target and pathway network (green represents the putative therapeutic active component, purple represents the putative therapeutic target, and pink represents the KEGG pathway).

In order to more directly understand the mechanism of Xiang Ju tablets in treating AR, we used the Cytoscape 3.7.0 Merge function to combine the “presumed therapeutic active ingredient-assumed therapeutic target” network, the presumed therapeutic target interaction network, and the involved KEGG pathways. The results showed that the interactions among active components, targets, and related pathways of Xiang Ju tablets played a therapeutic role (Figure 9).

## 4. Discussion

TCM compounds have multiple Chinese medicinal materials and ingredients and can treat diseases through multiple targets, channels, and links [12]. Fully clarifying the mechanism of action is difficult through traditional research methods. Network pharmacology can be used to study the correlation between the structure and function of many effective components of TCM through drug action networks, and a network of interaction between active component groups and multiple target proteins of the disease can be constructed and used to explain the mechanisms of TCM compounds at the molecular level [48, 49]. In this study, through network pharmacology and the use of multiple databases containing information such as compound protein biological information annotation, we constructed a network and performed target pathway enrichment analysis to systematically explore the mechanism of action of Xiang Ju tablets in the treatment of AR.

TCM has a long history in the treatment of sinusitis and has achieved good results. The Xiang Ju tablets produced by Shaanxi Xiangju Pharmaceutical Group Co., Ltd. are well known in the field of sinusitis treatment and have a considerable market share in China. Dong Qin brand Xiang Ju tablets produced by Shaanxi Xiangju Pharmaceutical Group Co., Ltd. are one of the only protected varieties of TCM. In this study, many methods based on network pharmacology were used to predict the targets, determine putative therapeutic targets, and build the network. This approach, combined with target prediction, was used to clarify the molecular synergistic effects of Xiang Ju tablets on AR. This approach provides clues to explore ethnopharmacology and/or herbal or even multidrug synergies. The potential pharmacological and molecular mechanisms of Xiang Ju tablets in treating AR were revealed through analysis of the putative therapeutic target network and biological functions.

In this study, network pharmacology was used to preliminarily screen and analyze the active component targets and mechanisms of Xiang Ju tablets, thus providing a basis for subsequent studies on the pharmacodynamic components and mechanisms of Xiang Ju tablets. However, this study has some shortcomings. First, owing to the limitation of screening conditions, only the main compounds in Xiang Ju tablets could be analyzed. Second, although a large number of targets and pathways can be screened through network pharmacology technology, these results must be verified by pharmacological experiments. Active compound of Xiang Ju tablets and the molecular mechanisms of its action on AR were predicted as a whole, and multiple potential therapeutic targets were explored, thus laying a good theoretical foundation for further experimental verification and providing directions for further investigations of its molecular mechanism.

## Figures and Tables

**Table 1:** Directly acting disease targets of Xiang Ju tablets and topological parameters.

No.	Gene name	Entry	Degree
1	CXCL8	P10145	25
2	IL1B	P01584	24
3	IL6	P05231	24
4	IL10	P22301	24
5	TNF	P01375	24
6	MMP9	P14780	23
7	IL4	P05112	23
8	ICAM1	P05362	22
9	IL13	P35225	21
10	CSF2	P04141	20
11	IL2	P60568	20
12	IL5	P05113	20
13	IFNG	P01579	19
14	FN1	P02751	19
15	IL1A	P01583	19
16	TGFB1	P01137	18
17	SPP1	P10451	16
18	SERPINE1	P05121	16
19	AHR	P35869	14
20	CCL11	P51671	14
21	CASP1	P29466	14
22	ALOX5	P09917	11
23	HIF1A	Q16665	11
24	NR3C1	P04150	9
25	PLAT	P00750	9
26	MS4A2	Q01362	4
27	TBXA2R	P21731	3
28	CHRM3	P20309	2
29	CHRM5	P08912	2
30	BCL2	P10415	1
31	ADRA2C	P18825	1
32	PIK3CG	P48736	0

**Table 2:** Information on 48 presumed therapeutic ingredients in Xiang Ju tablets acting directly on AR.

No.	Name	Compounds	Degree
1	MOL000098	Quercetin	19
2	MOL000008	Apigenin	12
3	MOL000006	Luteolin	9
4	MOL000511	Ursolic acid	7
5	MOL000422	Kaempferol	6
6	MOL000415	Rutinum	6
7	MOL000173	Wogonin	6
8	MOL011865	Rosmarinic acid	4
9	MOL000358	Beta-sitosterol	4
10	MOL002565	Medicarpin	3
11	MOL000378	7-O-Methylisomucronulatol	3
12	MOL012131	Isodihydrofutoquinol A	2
13	MOL005003	Licoagrocarpin	2
14	MOL004891	Shinpterocarpin	2
15	MOL004328	Naringenin	2
16	MOL003896	7-Methoxy-2-methyl isoflavone	2
17	MOL001689	Acacetin	2
18	MOL001484	Inermine	2
19	MOL001002	Ellagic acid	2
20	MOL000371	3,9-Di-O-methylnissolin	2
21	MOL000313	Galgravin	2
22	MOL013077	Decursin	1
23	MOL012125	Denudanolide C	1
24	MOL005792	{5-[2′(R)-Hydroxy-3′-methyl-3′-butenyl-oxy]furocoumarin}	1
25	MOL004978	2-[(3R)-8,8-Dimethyl-3,4-dihydro-2H-pyrano[6,5-f]chromen-3-yl]-5-methoxyphenol	1
26	MOL004959	1-Methoxyphaseollidin	1
27	MOL004941	(2R)-7-Hydroxy-2-(4-hydroxyphenyl)chromen-4-one	1
28	MOL004814	Isotrifoliol	1
29	MOL004793	Marmesine	1
30	MOL003588	Prangenidin	1
31	MOL002844	Pinocembrin	1
32	MOL002202	Tetramethylpyrazine	1
33	MOL002157	Wallichilide	1
34	MOL001956	Cnidilin	1
35	MOL001944	Marmesin	1
36	MOL001941	Ammidin	1
37	MOL001939	Alloisoimperatorin	1
38	MOL001792	DFV	1
39	MOL001749	ZINC03860434	1
40	MOL000513	Gallic acid	1
41	MOL000497	Licochalcone A	1
42	MOL000449	Stigmasterol	1
43	MOL000414	Caffeic acid	1
44	MOL000392	Formononetin	1
45	MOL000380	(6aR,11aR)-9,10-Dimethoxy-6a,11a-dihydro-6H-benzofurano[3,2-c]chromen-3-ol	1
46	MOL000354	Isorhamnetin	1
47	MOL000314	(2S,3S,4S,5S)-2,5-Bis(3,4-dimethoxyphenyl)-3,4-dimethyltetrahydrofuran	1
48	MOL000296	Hederagenin	1

## Data Availability

The data that support the findings of this study are openly available at http://lsp.nwu.edu.cn/tcmspsearch.php, https://string-db.org/, https://www.drugbank.ca/, https://db.idrblab.org/ttd/, http://www.disgenet.org/, and https://www.uniprot.org/.

## Supplementary Materials

Supplementary Materials There are eight supplementary material documents: (1) “Active ingredient information of Xiang Ju tablets” file: this file contains ingredient information of the 9 herbs in the prescribed Xiang Ju tablets, namely, *Platycarya strobilacea* Sieb. et Zucc (with seeds removed), Prunellae Spica, Chrysanthemi Indici flos, Astragali radix, Magnoliae Flos, Saposhnikoviae Radix, Angelicae dahuricae radix, Glycyrrhizae radix et rhizoma, and Chuanxiong Rhizoma. (2) “Allergic rhinitis related targets” file: this file contains information on 170 targets of allergic rhinitis. (3) “Biological process” file: this file contains biological process analysis results of 32 putative therapeutic targets obtained from the GO functional enrichment analysis using the Bioconductor database in RStudio software. (4) “Cell composition” file: this file contains cell composition analysis results of 32 putative therapeutic targets obtained from the GO functional enrichment analysis using the Bioconductor database in RStudio software. (5) “Composition information of Xiang Ju tablets” file: this file contains information on 976 components of 9 herbs in the Xiang Ju tablets. (6) “KEGG pathway analysis” file: this file contains signal pathway analysis results of 32 putative therapeutic targets obtained from the KEGG signal pathway enrichment analysis using the Bioconductor database in RStudio software. (7) “Molecular function” file: this file contains results of the molecular function analysis of the 32 putative therapeutic targets obtained from the GO functional enrichment analysis using the Bioconductor database in RStudio software. (8) “Target information” file: this file contains target information of 9 herbs in the Xiang Ju tablets.
